# Unexpected para-aortic venous migration of n-butyl cyanoacrylate during transarterial sac embolization for a type II endoleak: A technical pitfall

**DOI:** 10.1016/j.jvscit.2026.102514

**Published:** 2026-09-19

**Authors:** Kohei Hamamoto, Emiko Chiba, Soichiro Kojima, Mamoru Arakawa, Harushi Mori

**Affiliations:** a Department of Radiology, Jichi Medical University, Shimotsuke, Tochigi, Japan; b Department of Cardiovascular Surgery, Jichi Medical University, Shimotsuke, Tochigi, Japan

**Keywords:** Type II endoleak, Endovascular aneurysm repair, Transarterial embolization, N-butyl cyanoacrylate, Venous migration

## Abstract

We report an unusual case of para-aortic venous migration of n-butyl cyanoacrylate (NBCA) during transarterial sac embolization for a type II endoleak after endovascular aneurysm repair. Preprocedural computed tomography and angiography demonstrated no venous communication. During progressive NBCA injection, sequential opacification of the fourth lumbar vein and a para-aortic venous structure became apparent, prompting immediate cessation of embolization. The patient remained asymptomatic, and follow-up imaging demonstrated stable aneurysm size without complications. This case highlights a rare technical pitfall and emphasizes careful fluoroscopic monitoring during NBCA sac embolization despite initially negative imaging findings.

Type II endoleak (T2EL) is the most common endoleak following endovascular aneurysm repair (EVAR). Persistent T2EL associated with aneurysm sac enlargement may require intervention.^1^, ^2^, ^3^ Transarterial embolization (TAE) using liquid embolic agents such as n-butyl cyanoacrylate (NBCA) is an effective treatment, particularly when selective catheterization of all feeding and draining vessels is difficult.^1^^,^^4^, ^5^, ^6^, ^7^ However, venous migration of NBCA during T2EL embolization has rarely been described. We report a case of unexpected para-aortic venous migration of NBCA that became apparent only during progressive sac embolization despite negative preprocedural computed tomography (CT) and angiographic findings. The patient provided written informed consent for publication of this case report and accompanying images.

## Case report

A 74-year-old man underwent EVAR using an Excluder endograft (W. L. Gore & Associates) with concomitant coil embolization of the left internal iliac artery 15 years previously for an infrarenal abdominal aortic aneurysm measuring 45 × 45 mm (anteroposterior × transverse diameter), which had enlarged by 6 mm over the preceding 6 months. Follow-up contrast-enhanced CT demonstrated gradual aneurysm sac enlargement. Five years after EVAR, the aneurysm measured 45 × 50 mm, and a T2EL supplied by the right iliolumbar artery via the fourth lumbar artery was identified. The aneurysm subsequently enlarged to 46 × 62 mm 10 years after EVAR and 51 × 73 mm immediately before TAE. The patient’s medical history included hypertension, diabetes mellitus, thyroid cancer treated surgically 12 years earlier, and acute myocardial infarction 10 years previously. He had smoked five cigarettes per day for 30 years but had quit smoking 10 years earlier. His medications included clopidogrel and antihypertensive agents. Laboratory findings, including coagulation parameters, were within normal limits. As the patient preferred a minimally invasive approach, TAE was planned. Contrast-enhanced CT obtained 3 months before embolization demonstrated persistent T2EL supplied by the right iliolumbar artery through the fourth lumbar artery, with possible additional involvement of the median sacral artery (Fig 1, *A* and *B*), without an aortocaval or definite lumbar arteriovenous fistula. Under local anesthesia, a 5F sheath was inserted into the right common femoral artery. A 4F Cobra catheter (GlideCath; Terumo) and a 3F guiding sheath (Parent Plus 30; Medikit) were advanced coaxially into the right internal iliac artery. Angiography confirmed a T2EL through the iliolumbar and fourth lumbar arteries and also suggested possible involvement of the median sacral artery (Fig 1, *C*). A triaxial system consisting of a LEONIS MOVA High Flow microcatheter (Sumitomo Bakelite), a 1.9F Carnelian MARVEL microcatheter (Tokai Medical Products), and a 0.014-inch CHIKAI Black guidewire (Asahi Intecc) was advanced through the fourth lumbar artery into the aneurysm sac. No findings suggestive of guidewire or microcatheter perforation were observed. Selective angiography at each level during catheter advancement demonstrated no venous opacification or arteriovenous shunting. Sac angiography demonstrated neither venous drainage nor a contralateral-feeding artery (Fig 1, *D*). Because possible involvement of the median sacral artery was suggested by preprocedural CT and right internal iliac artery angiography, but selective access through the aneurysm sac or collateral pathways was technically difficult, NBCA was selected for sac packing rather than coil embolization of individual vessels. A 25% mixture of NBCA and Lipiodol was slowly injected under continuous fluoroscopic monitoring (Fig 2, *A* and *B*). After approximately 2 mL had been injected, the right fourth lumbar vein became opacified (Fig 2, *C*), and injection was temporarily suspended. After confirming no further progression, injection was cautiously resumed. Following an additional approximately 1 mL, another fine tubular structure extending ventrocaudally from the aneurysm sac became opacified (Fig 2, *D-F*). Unexpected venous migration of NBCA was suspected, prompting immediate cessation of injection. Completion angiography confirmed disappearance of the endoleak. Immediate noncontrast CT demonstrated NBCA-Lipiodol within the aneurysm sac, right fourth lumbar vein, and a fine tubular structure extending ventrocaudally from the aneurysm wall (Fig 3, *A-C*), without deposition in the vertebral body, spinal canal, inferior vena cava, or pulmonary arteries. Retrospective review of pre-EVAR and preprocedural CT demonstrated a tiny vessel extending from the aneurysm toward the inferior vena cava that corresponded anatomically to the NBCA-Lipiodol cast (Fig 3, *D-I*). The patient remained asymptomatic and was discharged home on postoperative day 2. At 3 months, CT demonstrated stable aneurysm size and persistent NBCA-Lipiodol casts.

**Fig 1:**
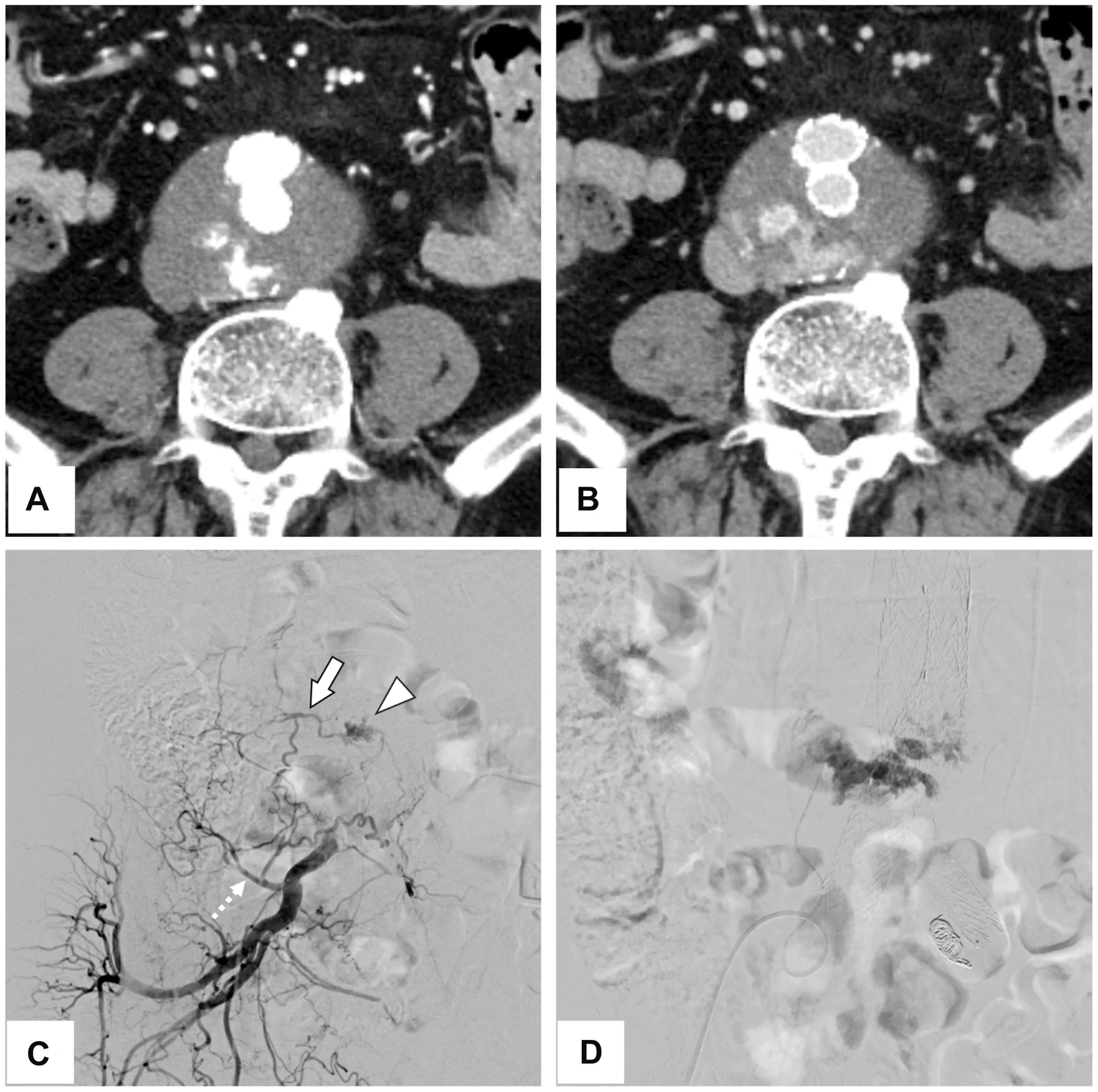
Preprocedural contrast-enhanced computed tomography (CT) and digital subtraction angiography. **A,** Arterial-phase CT demonstrates a type II endoleak (*arrowhead*). **B,** Venous-phase CT. **C,** Selective right internal iliac angiography demonstrates retrograde filling of the aneurysm sac through the iliolumbar artery (*dashed arrow*) and the right fourth lumbar artery (*arrow*), consistent with a type II endoleak (*arrowhead*). **D,** Sac angiography performed through the microcatheter demonstrates opacification of the aneurysm sac without visualization of venous drainage.

**Fig 2:**
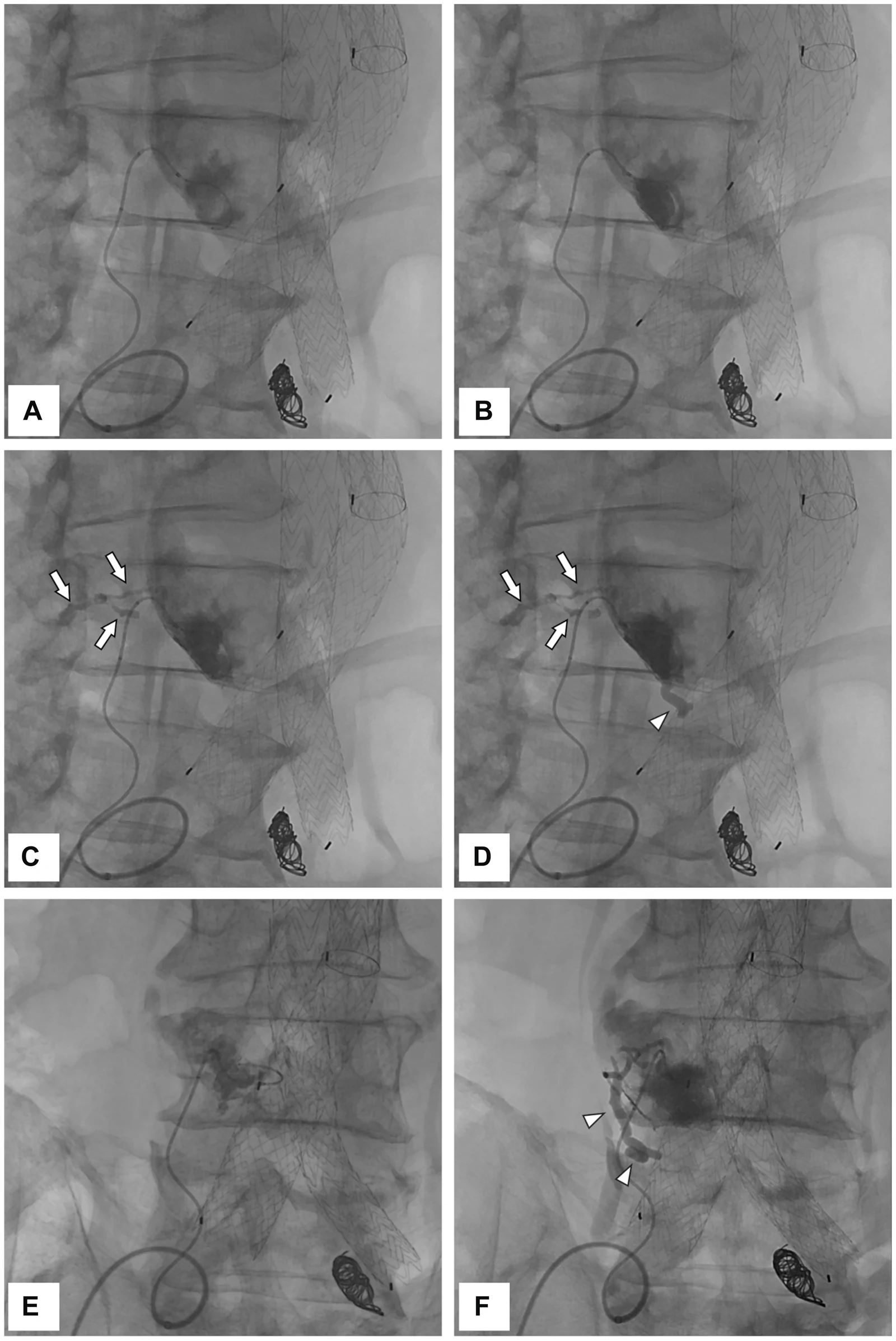
Sequential fluoroscopic images obtained during transarterial aneurysm sac embolization with n-butyl cyanoacrylate (NBCA). **A**-**D,** Right anterior oblique (RAO) view. **E** and **F,** Anteroposterior (AP) view. **A,** Before NBCA injection, residual contrast material is present within the aneurysm sac. **B,** During NBCA injection, the aneurysm sac becomes progressively filled with NBCA-Lipiodol. **C,** After approximately 2 mL of NBCA injection, a tubular structure extending dorsally from the aneurysm sac, consistent with the right fourth lumbar vein, becomes newly opacified (*arrows*). **D,** Following further NBCA injection, an additional tubular structure extending ventrocaudally from the aneurysm sac becomes newly opacified (*arrowhead*), suggesting unexpected para-aortic venous migration of NBCA. The injection was immediately discontinued. **E** and **F,** Comparison of AP fluoroscopic images obtained before **(E)** and after **(F)** embolization confirms newly developed opacification of the para-aortic venous structure (*arrowheads*).

**Fig 3:**
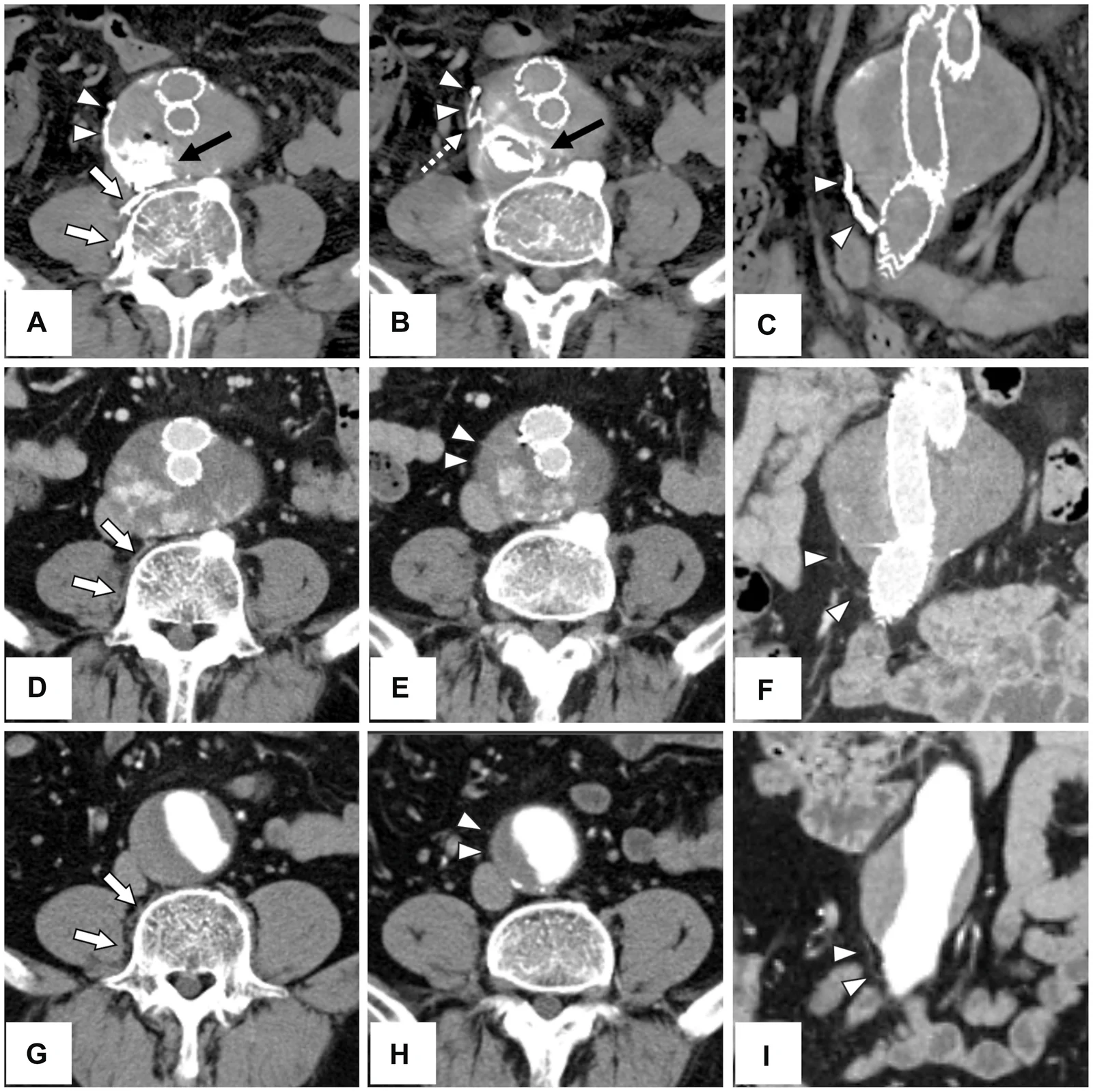
Postprocedural noncontrast computed tomography (CT) and comparison with preprocedural and pre-endovascular aneurysm repair (EVAR) contrast-enhanced CT. **A**-**C,** Noncontrast CT obtained immediately after embolization. **D**-**F,** Preprocedural contrast-enhanced CT. **G**-**I,** Contrast-enhanced CT obtained before EVAR. **A, B, D, E, G** and **H,** Axial images. **C, F** and **I,** Coronal images obtained at corresponding levels. Postprocedural CT demonstrates n-butyl cyanoacrylate (NBCA)-Lipiodol casts within the aneurysm sac (*black arrows*), the right fourth lumbar vein (*white arrows*), and a fine para-aortic venous structure extending from the right aspect of the aneurysm toward the ventrocaudal direction (*arrowheads*). In **(B)**, the NBCA-Lipiodol cast appears contiguous with the aneurysm wall (*dotted arrow*). The corresponding para-aortic venous structure is retrospectively identified on both the preprocedural CT **(E** and **F)** and the pre-EVAR CT **(H** and **I)**, where it is seen to communicate with the inferior vena cava.

## Discussion

TAE is a widely accepted minimally invasive treatment for persistent T2EL associated with aneurysm sac enlargement. Although the ideal therapeutic strategy is to occlude the endoleak cavity and all relevant inflow and outflow vessels, complete selective embolization is often difficult because T2ELs may involve multiple collateral pathways.^1^^,^^2^^,^^4^ Several studies have therefore emphasized the importance of embolizing the endoleak nidus or aneurysm sac, and liquid embolic agents such as NBCA can facilitate penetration into complex collateral channels that cannot be individually catheterized.^5^, ^6^, ^7^ However, clinical outcomes remain variable, and incomplete nidus or sac embolization may result in persistent endoleak or continued sac enlargement.^4^^,^^8^

This case demonstrates unexpected migration of NBCA into the lumbar and para-aortic veins that became apparent only during progressive sac embolization despite the absence of venous opacification on preprocedural angiography. Although the precise mechanism responsible for this phenomenon remains uncertain, progressive NBCA deposition may have altered local hemodynamics within the endoleak cavity: as preferential outflow channels became occluded, NBCA may have been redirected into a previously unapparent low-flow venous pathway. This interpretation is supported by the delayed appearance of venous filling only after continued sac embolization.

Retrospective CT demonstrated a pre-existing tiny vessel extending from the aneurysm toward the inferior vena cava and corresponding anatomically to the postoperative NBCA-Lipiodol cast. Although its precise nature could not be determined, it may have represented a small para-aortic venous tributary communicating with the lumbar venous system. Alternatively, NBCA may have traversed aneurysm wall or adventitial microvasculature before entering an enlarged draining vein. Neovascularization and remodeling of the adventitial vasa vasorum have been demonstrated in abdominal aortic aneurysms, providing a potential biological basis for this pathway, although this remains speculative.^9^, ^10^, ^11^, ^12^, ^13^ A lymphatic pathway represents another possible explanation; however, a venous structure was considered more likely because the vessel appeared to communicate with the inferior vena cava. Nevertheless, a lymphatic origin cannot be completely excluded.

Absence of NBCA-Lipiodol in the vertebral body or spinal canal on postprocedural CT makes migration through vertebral or radiculomedullary pathways unlikely. Although penetration of liquid embolic agents into draining veins is recognized during embolization of intracranial and spinal arteriovenous shunts,^14^ no arteriovenous shunt was demonstrated in this case. To our knowledge, sequential opacification of the lumbar and para-aortic veins only during progressive NBCA sac embolization for T2EL has not been previously reported.

This case emphasizes that negative preprocedural angiography does not exclude subsequent venous migration during sac embolization. Continuous fluoroscopic monitoring throughout NBCA injection and immediate cessation when unexpected venous opacification appears are essential to prevent migration into the central venous circulation. Careful review of preprocedural CT for subtle para-aortic venous structures may also help identify patients at potential risk.

## Conclusions

This case highlights unexpected para-aortic venous migration of NBCA during transarterial sac embolization for T2EL. Careful fluoroscopic monitoring is essential because venous migration may become apparent despite negative preprocedural CT and angiographic findings.

## Funding

None.

## Disclosures

None.

## References

[1] Chun J.Y., de Haan M., Maleux G., Osman A., Cannavale A., Morgan R. (2024). CIRSE standards of practice on management of endoleaks following endovascular aneurysm repair. Cardiovasc Intervent Radiol.

[2] Chen J.X., Stavropoulos S.W. (2020). Type 2 endoleak management. Semin Intervent Radiol.

[3] Avgerinos E.D., Chaer R.A., Makaroun M.S. (2014). Type II endoleaks. J Vasc Surg.

[4] Gallagher K.A., Ravin R.A., Meltzer A.J., Khan M.A. (2012). Midterm outcomes after treatment of type II endoleaks associated with aneurysm sac expansion. J Endovasc Ther.

[5] Yu H., Desai H., Isaacson A.J., Dixon R.G., Farber M.A., Burke C.T. (2017). Comparison of type II endoleak embolizations: embolization of endoleak nidus only versus embolization of nidus and branch vessels. J Vasc Interv Radiol.

[6] Ushijima Y., Asayama Y., Nishie A. (2019). Safety and efficacy of embolization using n-butyl cyanoacrylate for endoleaks after endovascular aortic repair. Interv Radiol (Higashimatsuyama).

[7] Fujitsuna R., Ueda T., Saito H. (2024). Initial outcomes of embolization for type II endoleak: comparison of n-butyl cyanoacrylate-ethiodized oil mixture with n-butyl cyanoacrylate-ethiodized oil-ethanol mixture. J Vasc Interv Radiol.

[8] Iwakoshi S., Ogawa Y., Dake M.D. (2023). Outcomes of embolization procedures for type II endoleaks following endovascular abdominal aortic repair. J Vasc Surg.

[9] Mäyränpää M.I., Trosien J.A., Fontaine V. (2009). Mast cells associate with neovessels in the media and adventitia of abdominal aortic aneurysms. J Vasc Surg.

[10] Sano M., Sasaki T., Hirakawa S. (2014). Lymphangiogenesis and angiogenesis in abdominal aortic aneurysm. PLoS One.

[11] Tanaka H., Zaima N., Sasaki T. (2013). Adventitial vasa vasorum arteriosclerosis in abdominal aortic aneurysm. PLoS One.

[12] Mulligan-Kehoe M.J., Simons M. (2014). Vasa vasorum in normal and diseased arteries. Circulation.

[13] Ritman E.L., Lerman A. (2007). The dynamic vasa vasorum. Cardiovasc Res.

[14] Maimon S., Luckman Y., Strauss I. (2016). Spinal dural arteriovenous fistula: a review. Adv Tech Stand Neurosurg.

